# Surface, mechanical and chemical properties of modified denture resin using natural biopolymer

**DOI:** 10.12669/pjms.39.6.7837

**Published:** 2023

**Authors:** Aftab Ahmed Khan, Abdulaziz Abdullah Alkhureif, Meshal Saeed Awaiyer, Leonel S J Bautista

**Affiliations:** 1 Aftab Ahmed Khan, PhD, MSc, M.Bioeth, B.D.S., Researcher, Dental Health Department, College of Applied Medical Sciences, King Saud University, Riyadh, Saudi Arabia; 2 Abdul Aziz Abdullah Alkhureif, PhD, MSc, B.Dent Tech, Professor, Dental Health Department, College of Applied Medical Sciences, King Saud University, Riyadh, Saudi Arabia; 3 Meshal Saeed Awaiyer, B.Dent Tech, Teaching Demonstrator, Dental Health Department, College of Applied Medical Sciences, King Saud University, Riyadh, Saudi Arabia; 4 Leonel S J Bautista, B.D.S., Researcher, Dental and Oral Rehabilitation Department, College of Applied Medical Sciences, King Saud University, Riyadh, Saudi Arabia

**Keywords:** Gum Arabic (GA), Denture resin, Poly Methyl Methacrylate (PMMA), Nanoindentation, Flexural strength

## Abstract

**Objective:**

This laboratory study determined the surface, mechanical and chemical properties of polymethyl methacrylate (PMMA) denture resin reinforced with micron-sized Gum Arabic (GA) powder in different weight ratios.

**Methods:**

This laboratory study was conducted at the Dental Health Department of the College of Applied Medical Sciences, King Saud University, Riyadh, Saudi Arabia from November 2022 to February 2023. Three experimental denture resins were prepared by incorporating GA powder in heat-polymerized PMMA powder using different wt.% (5, 10, and 20 wt.%). While pristine PMMA served as the control group. A total of ten bar-shaped specimens with dimensions of 65 mm × 10 mm × 3.5 mm were prepared for each study group. The surface properties (micro CT and SEM evaluation), mechanical properties (Nanohardness, elastic modulus and flexural strength) and chemical properties (FTIR) were conducted. The data were statistically analyzed using the one-way analysis of variance and Tukey’s post hoc tests (p<0.05).

**Results:**

The surface and bulk properties of experimental GA-reinforced PMMA resin materials deteriorated while the mechanical properties were also negatively altered using GA-based PMMA denture resin. A linear correlation was observed between weak mechanical properties and increasing wt.% of GA in denture resin.

**Conclusions:**

The incorporation of GA powder in denture resin might not be a viable option. The surface and mechanical properties of experimental PMMA composites were adversely affected compared to the control group.

## INTRODUCTION

There have been many materials introduced for the fabrication of denture bases. However, polymethyl methacrylate (PMMA) resin is a widely accepted and frequently used material.^1^ The low cost, simple handling and processing, and ease of polishing and repair are some of the desirable traits.^2^ However, this material lacks optimal mechanical requirements.^3^ Due to this, clinicians still encounter fractures of denture bases.^4^

Inadequate surface hardness and flexural strength of PMMA denture base material leave room for further development. Many other approaches have been experimented with for enhancing the mechanical properties such as utilizing substitute polymers i.e., polystyrene, polyethylene, and urethane dimethacrylate,^2^ copolymerization to change the chemical composition of PMMA.^5^ Nevertheless, the most commonly employed method is incorporating reinforcing additives including metal wire,^6^ filer particles^7^,^8^ and glass fibres.^9^ The approaches and strategies, however, did not work for one reason or another.

Gum Arabic (GA) is a naturally occurring polymer having antibacterial properties. It is also a non-toxic natural compound utilized in the sustained release of medications to deliver bioactive.^10^,^11^ GA could be a good substitute for traditional synthetic fibers used to reinforce denture bases. It is postulated that the addition of gum Arabic to PMMA may bond forces of the PMMA atoms. Additionally, this rubbery material has carboxyl groups which have the affinity to bind with the chemical groups of the PMMA.^12^

A recently published study showed that the binding affinity between GA and glass ionomer cement (GIC) is improved, thus enhancing the mechanical properties such as flexural strength, fracture toughness and tensile strength of the set GIC.^13^ In another study, GA powder was successfully reinforced into GIC for enhanced hardness, diametral tensile strength and compressive strength.^14^

Therefore, this laboratory study aimed to incorporate the varying weight ratios of micron-sized GA powder in a commercially available heat cure acrylic resin. The alternative hypothesis was that GA would increase the mechanical properties of denture base material.

## METHODS

This laboratory study was performed over three months at the Dental Health Department, College of Applied Medical Sciences, King Saud University Saudi Arabia, i.e., from November 2022 to February 2023. The study received exemption letter from the Institutional Review Board because no human or animal subjects were involved in this research. Pure GA (BonBalloon, KSA) in very a fine grade powder form (100-150 µm) was selected. The GA powder was soaked in 3-Methacryloxyproyltrimethoxysilane (MPS) using a commercially available dental silane coupling agent, ESPE™ Sil (3M ESPE, Seefeld, Germany). Thirty milliliter of MPS was used to agitate five grams of GA powder for two minutes. The extra solution was then decanted. Additionally, ethanol was used to rinse the GA powder twice. The GA powder was then dried for 24 hours at room temperature.

The dried GA powder was dispersed robustly in heat cure PMMA powder for five minutes using a vacuum mixer. The heat cure denture base resin (Interacryl Hot, Interdent, Opekarniska, Slovenia) was used with a powder: liquid ratio of 21g/10 ml according to the manufacturer’s recommendation. The mixture was manually stirred with a stainless steel spatula and a rubber bowl until it reached the dough-like stage.

Four different experimental groups with varying wt.% of GA powder incorporated in PMMA powder were fabricated: (A) Control group with 0 wt.% of GA powder, (B) 5 wt.% of GA, (C) 10 wt.% of GA, and (D) 20 wt.% of GA. Next, the dough was packed in a gypsum mould of a dental flask with inner dimensions of 65 mm **×**10 mm **×** 35 mm. A load of 100 N was applied to the flask for one minute to remove any excess resin material. For polymerization, the flask was placed in a water bath for eight hours at 73°C before being heated to 100°C for one hour again.^15^ After the polymerization process, the block was removed and sectioned according to the dimensions of ISO 1567:1999 specifications (65 mm **×**10 mm **×** 3.5 mm) for evaluating the flexural strength of denture base polymers^16^ using a precision diamond saw (Isomet 5000; Buehler Ltd, Lake Bluff, IL, USA) operating at 1600 revolutions per min while being cooled by water. To achieve polished surfaces, the specimens were ground with 320-grit silicon carbide paper. Before conducting any tests, the bar-shaped specimens were stored in a desiccator for 24 hours.

### Internal porosity evaluation:

A single bar-shaped specimen randomly selected from each study group was examined for porosity and agglomeration of GA powder using micro-computed tomography (Skyscan 1172, Bruker, Aartselaar, Belgium) at 100 kV voltage, 50 μA current, and 14.2 µm voxel size to characterize the agglomeration and pores in a three-dimensional structure. 360° rotation around the vertical axis was used for scanning the specimens. Using the porosity tool in the proprietary software (i.e., CTVol v.2.2.1, Bruker microCT), the overall porosity values were determined.

### Surface morphology evaluation

A randomly selected single specimen from each study group was evaluated for surface morphology using scanning electron microscopy (SEM) (JSM-6360LV, JEOL, Tokyo, Japan) in secondary electron mode at an accelerating voltage of 15 kV. SEM pictograms of the representative specimens were obtained using proprietary software at magnifications of 50 x.

### Surface topography test:

A micrometre-scale surface roughness of the specimens (n=10/group) was quantified with a non-contact surface profiler (Bruker Contour GT, Tucson, AZ, USA) as described previously.^17^ In short, the specimen’s surface was scanned at five different points using Vision64 (v 5.30) application software (Bruker, Campbell, CA, USA) to calculate the mean surface roughness (Ra, µm) value.

### Nanoindentation test

Nanoindentation tests were conducted on the specimens utilizing a nanomechanical instrument (UMT1, Bruker, CA, USA), fitted with a Berkovich diamond indenter nanotip (n=10/group). The experiments were performed at room temperature, employing loading and unloading rates of 0.5 mN/s and a 10 s dwell time. The maximum applied load was 20.0 mN.

### Fourier transform infrared (FTIR) spectroscopy

To identify the functional linkages and observe the compositional analysis, a randomly selected single specimen from each study group was measured for the FTIR spectrum. The targeted wavelength range was between 500 to 4000 cm^−1^ using a NICOLET iS5 spectrometer (Thermo Scientific, Massachusetts, USA). The system had a KBr beam splitter and a DTGS detector. With the aid of a monolithic diamond attenuated total reflectance (ATR iD7) accessory, spectra were recorded with a resolution of 2 cm^−1^.

### Three-point flexural strength test

The specimens for the flexural strength test were prepared per ANSI/ADA specification No.12 for denture base polymers with dimensions 65 × 10 × 2.5 mm^3^ (n=10/group). The specimens were subjected to a three-point bending flexural strength test using a universal testing machine (Model no. 3369 Instron; Canton, MA, USA). The proprietary software (Bluehill software version 2.6) of the testing machine recorded the flexural strength in megapascal (MPa). A load cell of 5 kN and a crosshead speed of 1mm/min was used for flexural strength evaluation.

### Statistical analysis

The obtained data were evaluated for descriptive and inferential statistics. The mean and standard deviation values were calculated in descriptive statistics while one-way analysis of variance (ANOVA) followed by *post hoc* Tukey’s HSD multiple comparison tests (p=0.05) were employed in inferential statistics. The statistical program, SPSS ver. 23.0 (IBM, New York, USA) was used for the statistical analyses.

## RESULTS

**Fig.1:**
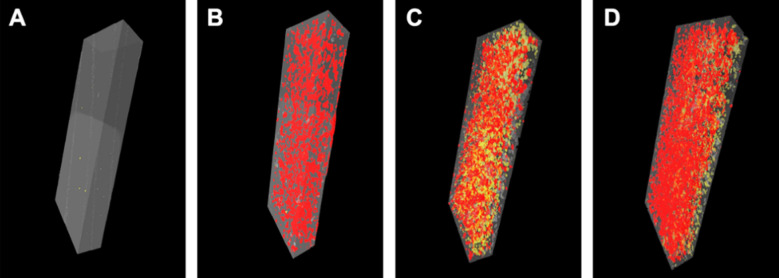
Micro-computed tomography analysis of GA particle distribution and gap/void formation in different study groups: (A) specimen of the control group, (B) specimen with 5 wt.% GA incorporated in denture base resin, (C) specimen with 10 wt.% GA incorporated in denture base resin and (D) specimen with 20 wt.% GA incorporated in denture base resin.

The 3D images of the study specimens are presented in Fig.1. Fig.1A shows the control specimen with sporadic yellow dots, presenting the voids in a specimen. While in Fig.1B, 1C and 1D, the red colour particles are denoting the GA powder incorporated to reinforce the denture base resin. The higher quantity of yellow area is evident in experimental groups, signifying the higher porosity in these groups compared to the control (i.e., without GA particles).

**Fig.2:**
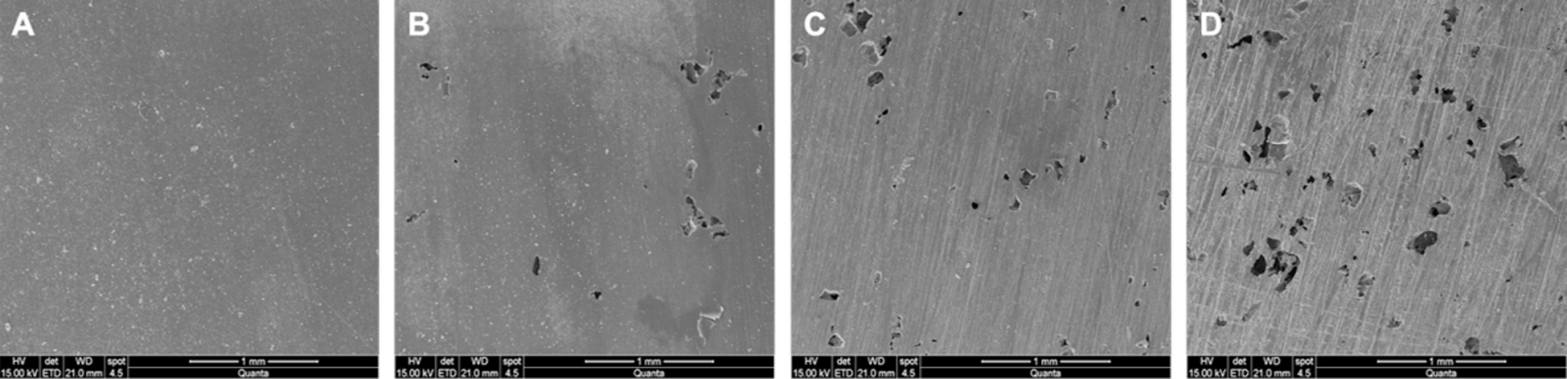
Analytical imaging of different study groups using SEM at 50X: (A) specimen of the control group, (B) specimen with 5 wt.% GA incorporated in denture base resin, (C) specimen with 10 wt.% GA incorporated in denture base resin, and (D) specimen with 20 wt.% GA incorporated in denture base resin.

Fig.2 depicts the SEM pictograms of the study groups. Fig.2A (i.e., control) demonstrates a smooth and clean surface without any voids. However, with the increasing filler loading, an increase in voids and porosity was observed. The visual analysis suggests the highest voids and porosity in 20 wt.% GA group (Fig.2D).

**Fig.3:**
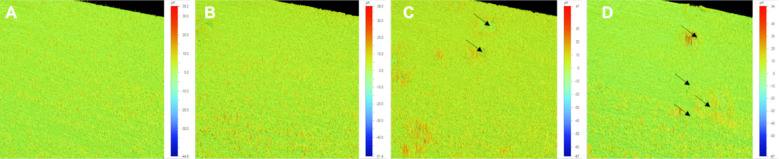
The 2D surface roughness profile images: (A) specimen of the control group, (B) specimen with 5 wt.% GA incorporated in denture base resin, (C) specimen with 10 wt.% GA incorporated in denture base resin, and (D) specimen with 20 wt.% GA incorporated in denture base resin.

Fig.3 illustrates the pictograms of the two-dimensional (2D) surface topography of the study specimens. Fig.3A (control) represented the least surface roughness among the tested group. However, increasing the GA filler loading resulted in increased surface roughness among the experimental groups. The deep valleys (in black arrows) were observed in Fig.3C and 3D.

The nanohardness (in GPa) and elastic modulus (in GPa) values of the tested groups are represented in Table-I. The available data suggest that the incorporation of GA powder not only reduces the nanohardness but also the elastic modulus of the denture base resin. The values of nanohardness and elastic modulus were observed as inversely proportional to wt.% of GA in denture base resin. The highest nanohardness and elastic modulus were observed in the control group (i.e., 0.27±0.05 GPa and 4.57±0.97 GPa, respectively). While 20 wt.% GA group showed the lowest nanohardness and elastic modulus values (i.e., 0.16±0.05 GPa and 3.12±0.70 GPa).

**Table-I:** Mean and standard deviation values of the nanohardness and elastic modulus of the study groups.

Group	Nanohardness (GPa)	Elastic modulus (GPa)
Control	0.27 ± 0.05^A^	4.57 ± 0.97
5 wt.% GA	0.24 ± 0.04	3.55 ± 0.70
10 wt.% GA	0.20 ± 0.03	3.22 ± 0.85
20 wt.% GA	0.16 ± 0.05^A^	3.12 ± 0.70

The surface roughness (in µm) and flexural strength (MPa) values of the tested groups are shown in Table-II. The incorporation of GA powder had a deleterious effect on the surface roughness of the specimens. The control group exhibited the least mean surface roughness value, i.e., 1.77±0.27 µm while 20 wt.% GA group demonstrated the highest, i.e., 3.45±0.61 µm. The details are presented in Table-II.

**Table-II:** Mean and standard deviation values of the surface roughness and flexural strength of the study groups.

Group	Surface roughness (µm)	Flexural strength (MPa)
Control	1.77 ± 0.27^A,B^	111.94 ± 4.24^A,B,C^
5 wt.% GA	2.39 ± 0.27^C^	88.99 ± 11.24^A,D,E^
10 wt.% GA	2.76 ± 0.21^A^	56.94 ± 7.92^B,D^
20 wt.% GA	3.45 ± 0.61^B,C^	54.42 ± 9.67^C,E^

The FTIR spectra of the study groups is presented in Fig-IV. The significant signals between 2800–2950 cm^-1^ were due to C–H vibrations. While the stretching vibrations of the ester carbonyl C=O between 1700-1750 cm^-1^, CH_2_ aromatic group in the range 1400-1450 cm^-1^, the C-O deformation between 1150-1200 cm^-1^, and the C-O-C vibration at 1149 cm^-1^. The original PMMA’s structure was unaffected by the inclusion of different wt.% GA.

**Fig.4:**
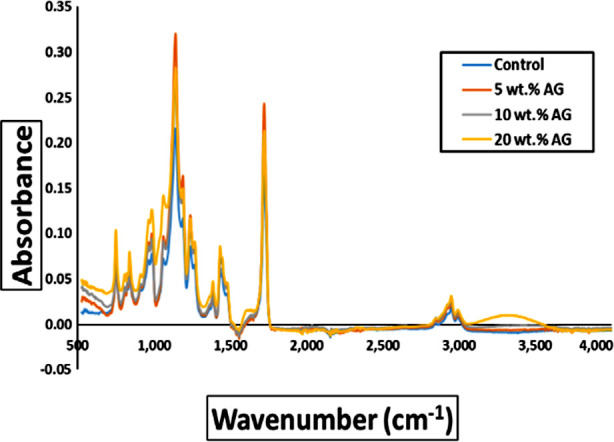
Spectroscopy analysis of pure and reinforced PMMA resin using different wt.% of GA.

## DISCUSSION

The hypothesis of this investigation is rejected: incorporation of varying wt.% of GA powder revealed a deleterious effect on the tested properties. The surface topography data suggest incorporation of GA powder increased the surface roughness of the PMMA composite, and the deleterious effect on surface roughness had a direct relationship with the wt.% of GA powder incorporated in PMMA. The higher filler loading as well as the poor distribution of GA powder in resin acrylic might have caused increased surface roughness.^18^ Although GA powder (80-150 μm in size) was silanized before mixing in PMMA powder, clustering of the powder might have occurred in set acrylic resin, causing increased surface roughness in experimental groups.^18^ Filler loading of more than 5wt% leads to agglomeration and cluster formation.^19^-^21^ Additionally, due to poor bonding between GA and PMMA resin, protrusion of GA from the specimen composite surface during PMMA composite fabrication might have increased the surface roughness among the experimental composites.^3^

Hardness is a vital property that can determine suitable material for a denture base.^22^-^24^ The nanoindentation data strongly suggests that the addition of GA powder had a deleterious effect on enhancing the nanohardness and elastic modulus of the GA-based composite resins. The decrease in properties hereinbefore described might be attributed to the plasticizing impact of GA, which affects the nanohardness and elastic properties of the acrylic denture resin.^1^ Also, the elastic modulus of polymeric composite is influenced by many reinforcement and polymer-related variables. Aspect ratio, particle alignment, clustering, and interphase are a few of them, also the particle loading (wt.%).^25^ The intrinsic bond strength between PMMA atoms in the lattice structure might had affected due to critical value of GA powder content. Hence, decreased adhesion between GA powder and PMMA polymer. Additionally, the higher filler loading in denture resin might have affected the elastic modulus.

Although the GA powder was silanized before mixing in PMMA powder, the decrease in flexural strength with the rate of GA powder addition could be due to the poor adhesion of GA powder with the PMMA matrix. Also, the use of GA powder created intrinsic voids (as shown in Fig.1) and surface voids (as shown in Fig.2) in the PMMA matrix. These voids could be due to the entrapment of gas during PMMA curing^26^, possibly the chemical reactions of GA powder with PMMA matrix at elevated temperatures. The formation of voids could also be due to the hand mixing of GA powder incorporated in PMMA powder with the MMA liquid. Additionally, the sonication and degassing of GA powder were not executed before incorporation. Hence, the formation of micro- and macro-voids in GA-based PMMA composites. Consequently, the flexural strength of the created composites exhibited a decreased nature when the GA proportion is increased. Due to increased load, the void grew and voids’ coalescence ensued, resulting in brittle-like failure.

The SEM images confirmed the presence of space/voids on the surface of the experimental composites. Whereas, microCT images further affirmed the formation of voids in the bulk materials. Here it is notable that tiny bubbles, during the polymerization process, grow larger either through amalgamation or expansion in an exothermic reaction.^27^ During mixing, bubbles that are imperceptible to the human eye suddenly become visible after curing. While the chemical analysis proved that the reinforcing GA powder did not affect the structure of the original PMMA.

Laboratory studies often use controlled and simplified conditions that might not fully mimic the complex oral environment. Laboratory studies often focus on evaluating one specific variable or material property at a time. In future research, it would be appropriate to use low filler loading of GA powder in PMMA resin. Additionally, improved silane or other surface treatments should be considered to improve the bonding between the GA and acrylic. Accelerated aging studies to assess the durability and stability of GA-modified denture acrylic over extended periods would be interesting also.

## CONCLUSION

Considering the outcome of this laboratory findings, it can be concluded that the addition of GA powder increased the surface topography of the GA-reinforced PMMA composite. Nanohardness, elastic modulus and flexural strength of the PMMA composites were compromised with the increasing wt.% of GA powder. GA powder might not be chemically compatible with the PMMA denture resin.

### Authors’ Contribution:

**AAK** performed data collection, data interpretation, and manuscript writing and is responsible for the accuracy and integrity of the research.

**AAA** conceived, supervised the study project and approval of manuscript writing.

**MSA** did statistical analysis, tabulation of the data, revision.

**LSJB** Specimens test, formatting and editing of the final draft.
